# Assessment of 2-Year Neurodevelopmental Outcomes in Extremely Preterm Infants Receiving Opioids and Benzodiazepines

**DOI:** 10.1001/jamanetworkopen.2021.15998

**Published:** 2021-07-07

**Authors:** Mihai Puia-Dumitrescu, Bryan A. Comstock, Sijia Li, Patrick J. Heagerty, Krystle M. Perez, Janessa B. Law, Thomas R. Wood, Semsa Gogcu, Dennis E. Mayock, Sandra E. Juul

**Affiliations:** 1 Division of Neonatology, Department of Pediatrics, University of Washington, Seattle; 2 Department of Biostatistics, University of Washington, Seattle; 3 Division of Neonatology, Department of Pediatrics, Wake Forest University School of Medicine, Winston-Salem, North Carolina

## Abstract

**Importance:**

Extremely preterm (EP) infants frequently receive opioids and/or benzodiazepines, but these drugs’ association with neurodevelopmental outcomes is poorly understood.

**Objectives:**

To describe the use of opioids and benzodiazepines in EP infants during neonatal intensive care unit (NICU) hospitalization and to explore these drugs’ association with neurodevelopmental outcomes at 2 years’ corrected age.

**Design, Setting, and Participants:**

This cohort study was a secondary analysis of data from the Preterm Erythropoietin Neuroprotection (PENUT) Trial, which was conducted among infants born between gestational ages of 24 weeks, 0 days, and 27 weeks, 6 days. Infants received care at 19 sites in the United States, and data were collected from December 2013 to September 2016. Data analysis for this study was conducted from March to December 2020.

**Exposures:**

Short (ie, ≤7 days) and prolonged (ie, >7 days) exposure to opioids and/or benzodiazepines during NICU stay.

**Main Outcomes and Measures:**

Cognitive, language, and motor development scores were assessed using the Bayley Scales of Infant Development–Third Edition (BSID-III).

**Results:**

There were 936 EP infants (448 [48%] female infants; 611 [65%] White infants; mean [SD] gestational age, 181 [8] days) included in the study, and 692 (74%) had neurodevelopmental outcome data available. Overall, 158 infants (17%) were not exposed to any drugs of interest, 297 (32%) received either opioids or benzodiazepines, and 481 (51%) received both. Infants exposed to both had adjusted odds ratios of 9.7 (95% CI, 2.9 to 32.2) for necrotizing enterocolitis and 1.7 (95% CI, 1.1 to 2.7) for severe bronchopulmonary dysplasia; they also had a longer estimated adjusted mean difference in length of stay of 34.2 (95% CI, 26.2 to 42.2) days compared with those who received neither drug. After adjusting for site and propensity scores derived for each exposure category, infants exposed to opioids and benzodiazepines had lower BSID-III cognitive, motor, and language scores compared with infants with no exposure (eg, estimated difference in mean scores on cognitive scale: −5.72; 95% CI, –8.88 to –2.57). Prolonged exposure to morphine, fentanyl, midazolam, or lorazepam was associated with lower BSID-III scores compared with infants without exposure (median [interquartile range] motor score, 85 [73-97] vs 97 [91-107]). In contrast, BSID-III scores for infants with short exposure to both opioids and benzodiazepines were not different than those of infants without exposure.

**Conclusions and Relevance:**

In this study, prolonged combined use of opioids and benzodiazepines was associated with a risk of poorer neurodevelopmental outcomes as measured by BSID-III at 2 years’ corrected age.

## Key Points

**Question:**

Is there an association between 2-year neurodevelopmental outcomes and use of opioids and/or benzodiazepines for sedation and analgesia in extremely preterm infants?

**Findings:**

In this cohort study of 936 infants born at gestational ages between 24 weeks, 0 days, and 27 weeks, 6 days, those exposed to both opioids and benzodiazepines for more than 7 days were more likely to have increased in-hospital morbidities, prolonged length of stay, and lower BSID-III cognitive, motor, and language scores at 2 years’ corrected age compared with infants without exposure or those exposed to opioids or benzodiazepines alone.

**Meaning:**

In this study, the use of opioids and/or benzodiazepines among extremely preterm infants was associated with adverse effects on 2-year neurodevelopmental outcomes.

## Teaser

This cohort study describes the use of opioids and benzodiazepines in extremely preterm infants during neonatal intensive care unit hospitalization and explores these drugs’ association with neurodevelopmental outcomes at 2 years’ corrected age.

## Introduction

The use of analgesia and/or sedation for the smallest premature infants with the most severe illness is at the discretion of the medical team, and the overall treatment goals are to mitigate pain, agitation, and discomfort. The American Academy of Pediatrics (AAP) recognizes that neonates at greatest risk of neurodevelopmental impairment as a result of preterm birth are also those most likely to be exposed to the greatest number of painful stimuli in the neonatal intensive care unit (NICU).^1^ The management of discomfort and agitation remains an integral part of the care for infants in the NICU, as acute and chronic pain result in heightened stress, hyperalgesia-associated reductions in white matter and corticospinal tract fractional anisotropy, and long-term behavioral changes.^2,3,4,5,6^ Thus, the AAP recommends pharmacologic and nonpharmacologic therapies for the prevention of pain associated with routine minor procedures as well as measures for minimizing pain associated with surgery and other major procedures.^1,7^ This recommendation is in stark contrast to prior practice, when infants received no analgesia during surgical procedures until the 1980s.^8,9^ Routine administration of opioids and benzodiazepines in extremely preterm (EP) infants is not recommended due to concerns regarding safety, efficacy, and potential long-term consequences.^1,7^

The role of analgesia and/or sedation is even less clear in the smallest infants, particularly during critical stages of brain development.^10^ Opioid analgesics have been associated with the risk of smaller cortical and cerebellar volumes as well as lower cognitive and motor scores on standardized neurodevelopmental tests,^11,12,13^ while benzodiazepines have been shown to cause neuroapoptosis with increased risk of brain injury in developing rodents.^14,15^

The long-term neurodevelopmental implications of prolonged therapy with opioids, benzodiazepines, and/or a combination of the 2 drugs in EP infants are poorly understood. Given the lack of data regarding the outcomes of these infants, using data from a large multicenter cohort of EP infants, we performed an analysis to describe the use of these drugs in the NICU and then to characterize their association with 2-year neurodevelopmental outcomes.

## Methods

### Data Source and Study Population

All infants born between 24 weeks, 0 days, and 27 weeks, 6 days, of gestation between December 2013 and September 2016 without known life-threatening anomalies, chromosomal anomalies, disseminated intravascular coagulopathy, twin-to-twin transfusion, a hematocrit level greater than 65%, hydrops fetalis, or known congenital infection and enrolled in the Preterm Erythropoietin Neuroprotection (PENUT) Trial (NCT01378273) were eligible for this study.^16^ The PENUT trial was approved by the institutional review board at each participating site. Parental consent was obtained before or after birth, as permitted by the institutional review board at each site. This study followed the Strengthening the Reporting of Observational Studies in Epidemiology (STROBE) reporting guideline.

We collected data about maternal characteristics, pregnancy, and delivery as well as infant characteristics, including exposure to medications and comorbidities during their NICU stay. At 22 to 26 months’ corrected age, the infants were examined and evaluated by certified examiners who assessed cognitive, motor, and language development with the Bayley Scales of Infant Development–Third Edition (BSID-III).

The analgesics and sedatives of interest for this study were grouped into 2 classes: opioids (morphine or fentanyl) and benzodiazepines (midazolam, diazepam, clonazepam, or lorazepam). Exposure to the medications of interest was defined as no exposure if there were no documented or reported medications of interest administered at any time during the NICU stay (any day from birth to death or hospital discharge), short exposure if an infant received the medications of interest for 7 or fewer days, and prolonged exposure if the infant received a cumulative exposure to the medications of interest for more than 7 days. We characterized the exposure variable as the cumulative number of days each infant was exposed to opioids and/or benzodiazepines. The exposure was captured as yes or no on any given day, without accounting for concurrent administration of more than 1 medication of interest. When describing the cohort characteristics, we excluded medication data if exposure occurred within 3 days after a surgical procedure. For analysis of an association between the use of opioids and/or benzodiazepines and 2-year outcomes, we included all days of exposure, including postoperative use.

### Statistical Analysis

We used descriptive statistics and graphic illustrations to describe the demographic and baseline maternal and infant characteristics. We aimed to evaluate the association between opioid and/or benzodiazepine exposure and neurodevelopmental outcomes measured by BSID-III scores. We used generalized estimating equations^17^ with robust standard errors and an exchangeable working covariance structure as the statistical model to appropriately account for potential association of outcomes for same-birth siblings. Specifically, we performed linear multivariate analyses using each of the 3 BSID-III scores (ie, cognitive, motor, and language) as the outcome, stratified by duration of exposure (ie, no exposure, short term, long term) to all medications of interest combined, opioids and benzodiazepines individually, and the 2 most commonly used drugs in each class (ie, fentanyl, morphine, lorazepam, and midazolam), and adjusted for PENUT treatment group (erythropoietin or placebo), study site, and gestational age (GA) in weeks. In addition, we adjusted for the linear predictors of 2 propensity scores to account for potential bias due to confounding by indication. Given that the exposure variable was 3-level and ordinal, we defined the propensity scores to be: (1) the probability of receiving any exposure (1-7 days or >7 days) vs none and (2) the probability of receiving prolonged exposure (>7 days) vs no exposure or 1 to 7 days of exposure. The 2 propensity scores for each infant were derived by fitting a logistic regression model using exposure as the outcome and adjusting for the following potential confounders: treatment assignment, GA at birth in weeks, infant weight, infant sex, multiple gestation (yes or no), maternal education level (high school or less, some college, or BA/BS or greater), maternal race (Black, other [American Indian or Alaska Native, Asian, and Native Hawaiian or other Pacific Islander], or White), maternal hypertension (yes or no), chorioamnionitis (yes or no), maternal iron supplementation during pregnancy (yes or no), prenatal steroids (yes or no), tobacco use (yes or no), alcohol use (yes or no), prescription drug use (yes or no), recreational drug use (yes or no), Apgar score at 5 minutes, and baseline intraventricular hemorrhage (IVH; no, mild [grade I-II], or severe [grade III-IV]). We considered adjustment for comorbidities, such as IVH at week 36, necrotizing enterocolitis (NEC), and/or bronchopulmonary dysplasia (BPD), but these events largely occur after opioid exposure and therefore would not be confounders. Infants with missing data (53 [6%] for IVH; 29 [3%] for race; 11 [1%] for prescription drug use; 3 [<1%] for 5-minute Apgar; 1 [<1%] for prenatal magnesium sulfate; and 1 [<1%] for drug use) were imputed using multiple imputation method (mice package in R).^18^

We reported the estimated average differences in BSID-III scores between groups characterized by days of exposure, along with their 95% CIs and corresponding *P* value. Statistical significance was set at *P* < .05, and all tests were 2-tailed. All analyses were conducted using the R statistical package version 3.6.1 (R Project for Statistical Computing).

## Results

### Cohort Characteristics

A total of 936 infants born at 19 sites were included in this analysis. Of these, 448 (48%) were female infants, 611 (65%) were White infants, and mean [SD] GA was 181 (8) days. Overall, 481 infants (51%) were exposed to both opioids and benzodiazepines, 297 (32%) were exposed to opioids or benzodiazepines, and 158 (17%) were not exposed to either class of medications of interest (Table). Exposure to benzodiazepines alone was uncommon, with only 20 infants (2%) in this category. Infants exposed to both opioids and benzodiazepines tended to be smaller and born earlier than infants without exposure and those exposed to only 1 of the 2 classes of medications. The mode of delivery was not significantly different between the groups, with 285 (30%) infants born vaginally. Maternal characteristics (except for maternal education and prescription drug use) were not statistically different between the groups.

**Table.** Demographic Characteristics and In-Hospital Morbidities by Opioid and Benzodiazepine Exposure Categories

Characteristic	Patients, No. (%)			*P* value^d^
	Neither (n = 158)^a^	Either (n = 297)^b^	Both (n = 481)^c^	
**Maternal and pregnancy characteristics**				
Multiple gestation				
No	106 (15)	219 (32)	368 (53)	.06
Yes	52 (21)	78 (32)	113 (46)	
Maternal education				
≤High school	51 (17)	112 (37)	144 (47)	.005
Some college	59 (21)	90 (32)	136 (48)	
≥BA/BS degree	36 (16)	70 (30)	126 (54)	
Not reported	12 (11)	25 (22)	75 (67)	
Maternal race				
Black	42 (18)	76 (32)	122 (51)	.48
White	105 (17)	191 (31)	315 (52)	
Other^e^	10 (18)	21 (38)	25 (45)	
Maternal hypertension				
No	130 (18)	224 (30)	389 (52)	.12
Yes	28 (15)	73 (38)	92 (48)	
Chorioamnionitis				
No	144 (18)	251 (31)	419 (52)	.13
Yes	14 (12)	46 (38)	62 (51)	
Prenatal steroids				
No	8 (10)	27 (35)	42 (55)	.27
Yes	148 (18)	267 (32)	427 (51)	
Tobacco use				
No	139 (17)	260 (32)	425 (52)	.94
Yes	19 (17)	37 (33)	56 (50)	
Alcohol use				
No	153 (17)	289 (32)	474 (52)	.32
Yes	5 (25)	8 (40)	7 (35)	
Prescription drugs				
No	109 (17)	215 (34)	307 (49)	.04
Yes	46 (16)	79 (27)	169 (58)	
Recreational drugs				
No	152 (17)	277 (32)	444 (51)	.27
Yes	6 (10)	20 (32)	36 (58)	
Delivery method				
Cesarean	15 (19)	22 (28)	41 (53)	.79
Emergency cesarean	93 (16)	179 (31)	301 (53)	
Vaginal	50 (18)	96 (34)	139 (49)	
**Infant characteristics**				
5-min Apgar score, mean (SD)	6.8 (1.7)	6.2 (2.2)	6.0 (2.2)	<.001
Treatment group				
Placebo	69 (15)	142 (31)	249 (54)	.18
Erythropoietin	89 (19)	155 (33)	232 (49)	
Gestational age at birth, wk				
24	17 (7)	66 (28)	149 (64)	<.001
25	22 (9)	72 (29)	151 (62)	
26	51 (23)	72 (33)	98 (44)	
27	68 (29)	87 (37)	83 (35)	
Birth weight, mean (SD), g	894 (181)	806 (185)	765 (182)	<.001
Sex				
Male	74 (15)	146 (30)	268 (55)	.07
Female	84 (19)	151 (34)	213 (48)	
Intraventricular hemorrhage grade, first day of life				
No hemorrhage	119 (17)	230 (33)	346 (50)	.40
Grade I-II	20 (14)	43 (31)	78 (55)	
Grade III-IV	7 (15)	11 (23)	29 (62)	
Missing	12 (23)	13 (25)	28 (53)	
Intubation				
>12 h (n = 820)	100 (12)	251 (31)	469 (57)	<.001
>1 wk (n = 580)	37 (6)	156 (27)	387 (67)	<.001
Vasoactive drugs in the first 72 h^f^	14 (6)	58 (24)	171(70)	<.001
**In-hospital morbidities**				
Severe retinopathy of prematurity (n = 72)	1 (1)	12 (4)	57 (12)	<.001
Intraventricular hemorrhage grade III-IV (n = 124)	5 (3)	27 (9)	92 (19)	<.001
Necrotizing enterocolitis				
All (n = 99)	3 (2)	28 (9)	68 (14)	<.001
Surgical (n = 65)	0	18 (6)	47 (10)	<.001
Patent ductus arteriosus ligation (n = 106)	0	23 (8)	83 (17)	<.001
Severe bronchopulmonary dysplasia (n = 333)	48 (30)	99 (33)	186 (39)	.028
Tracheostomy (n = 22)	0	3 (1)	19 (4)	.003
CPAP/HFNC at 36 wk (n = 182)	15 (9)	48 (16)	119 (25)	<.001
Noninvasive nasal ventilation at 36 wk (n = 41)	4 (3)	13 (4)	24 (5)	.38
Ventilator dependency at 36 wk (n = 56)	3 (2)	12 (4)	41 (9)	.002
Length of stay, mean (SD), d	91 (58)	104 (35)	124 (26)	<.001

Abbreviations: CPAP, continuous positive airway pressure; HFNC, high-flow nasal cannula.

^a^ No exposure to any opioids or benzodiazepines.

^b^ Exposure to opioids or benzodiazepines.

^c^ Exposure to opioids and benzodiazepines.

^d^ Differential baseline characteristics and in-hospital morbidities among different exposure groups were tested via either χ^2^ test for categorical variables or *t* test for continuous variables.

^e^ Other racial groups included American Indian or Alaska Native, Asian, and Native Hawaiian or other Pacific Islander.

^f^ Vasoactive drugs included epinephrine, dopamine, dobutamine, vasopressin, and norepinephrine.

Infants exposed to opioids and benzodiazepines had more comorbidities compared with those exposed to 1 class or not exposed (Table). Of 72 infants with severe retinopathy of prematurity, 57 (79%) were exposed to both drug classes; of 124 with grade III or IV IVH, 92 (74%) were exposed to both drug classes; of 99 with any NEC, 68 (69%) were exposed to both drug classes; of 106 with patent ductus arteriosus ligation, 83 (78%) were exposed to both drug classes; and of 333 with severe BPD, 186 (56%) were exposed to both. Infants exposed to both classes had more comorbidities, with adjusted odds ratios of 4.5 (95% CI, 1.7-11.6) for IVH, 9.7 (95% CI, 2.9-32.2) for NEC, 1.7 (95% CI, 1.1-2.7) for severe BPD, and 7.7 (95% CI, 1.7-33.1) for retinopathy of prematurity compared with those not exposed. Nearly all infants who were receiving ventilator support at 36 weeks’ postmenstrual age (53 of 56 infants [95%]) were exposed to either or both classes of medications of interest. Infants exposed to both had longer hospital stays compared with infants exposed to either class of medications alone or infants with no exposure (mean [SD], 124 [50] days vs 104 [45] days and 91 [25] days, respectively; *P* < .001). Estimated adjusted mean difference in length of stay was 1.4 (95% CI, 0.9-2.3) days for infants receiving either opioids or benzodiazepines and 34.2 (95% CI, 26.2-42.2) days for infants exposed to both compared with infants with no exposure.

### Exposure to Medications of Interest

The total days of exposure to opioids or benzodiazepines ranged from 0 to 379 days (median [IQR], 4 [1-26] days), with fentanyl ranging from 0 to 173 days (median [IQR], 1 [0-5] days), morphine from 0 to 204 days (median [IQR], 0 [0-9] days), midazolam from 0 to 75 days (median [IQR], 0 [0-1] days), and lorazepam from 0 to 259 days (median [IQR], 0 [0-4] days). Length of stay ranged from 50 to 442 days for the infants who survived to discharge.

Among the 778 infants exposed to opioids in this cohort, 590 (76%) received fentanyl, and 442 (57%) received morphine. Benzodiazepines were given to 501 infants, with 325 (65%) receiving midazolam and 320 (64%) receiving lorazepam during their NICU stay. Diazepam and clonazepam were rarely used, with 8 infants (2%) and 2 infants (<1%), respectively. The median (interquartile range [IQR]) duration of exposure for morphine, fentanyl, midazolam, and lorazepam was 10 (2-26) days, 3 (1-11) days, 3 (1-9) days, and 14 (4-34) days, respectively.

**Figure 1.**
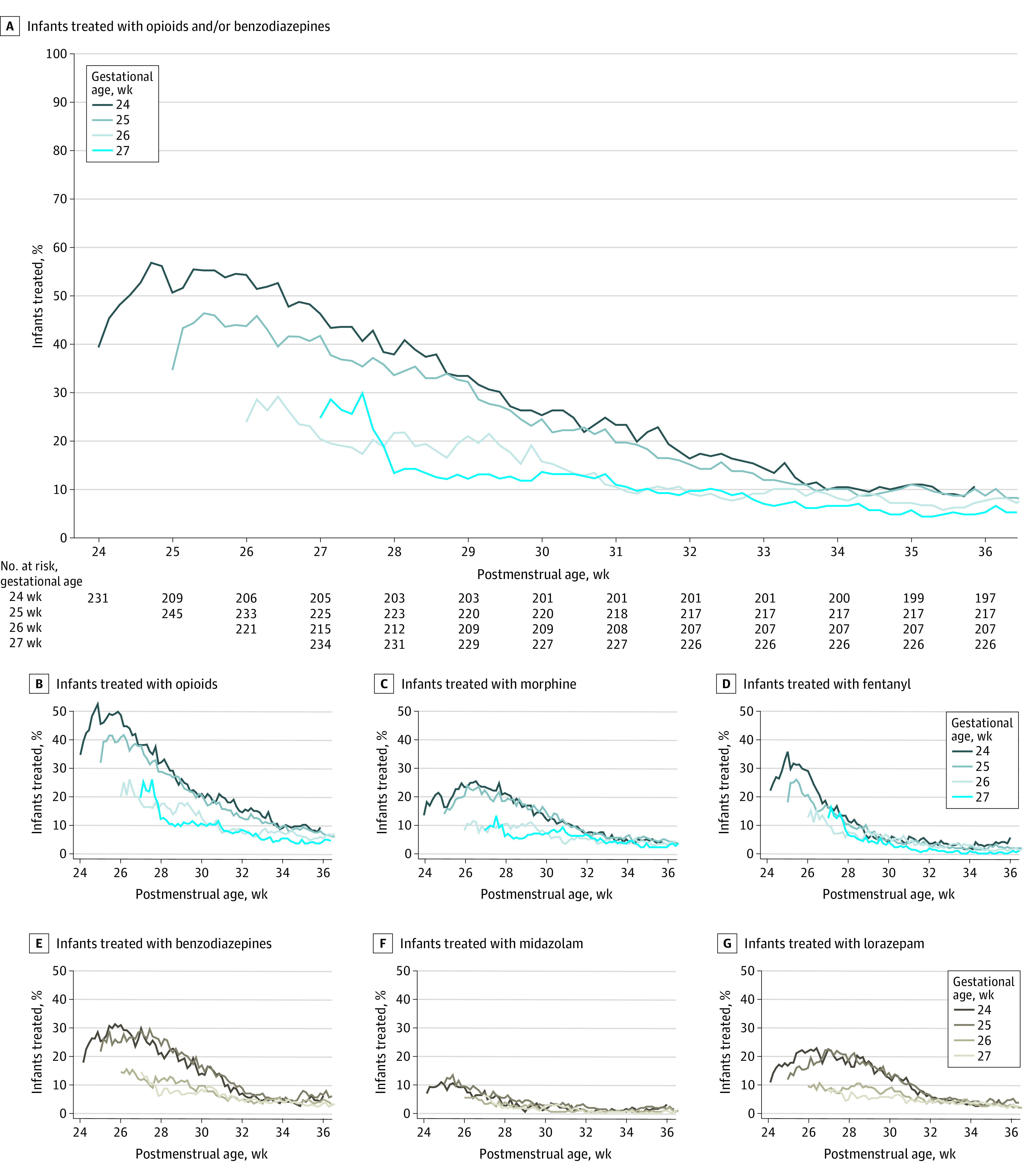
Exposure to Opioids and/or Benzodiazepines Over Time, Stratified by Gestational Age at Birth For each exposure of interest, the daily prevalence of exposure (defined as the number of exposed infants divided by the number that remained alive and in hospital) was plotted for each gestational age by postmenstrual age until 36 weeks.

Infants born at 24 to 25 weeks’ GA had earlier and higher overall exposure to opioids and benzodiazepines compared with infants born at 26 to 27 weeks’ GA (Figure 1). Of 203 infants with 24 weeks’ GA, 78 (38.4%) were receiving opioids and benzodiazepines at 28 weeks’ postmenstrual age compared with 30 of 227 infants (13.2%) with 27 weeks’ GA.

**Figure 2.**
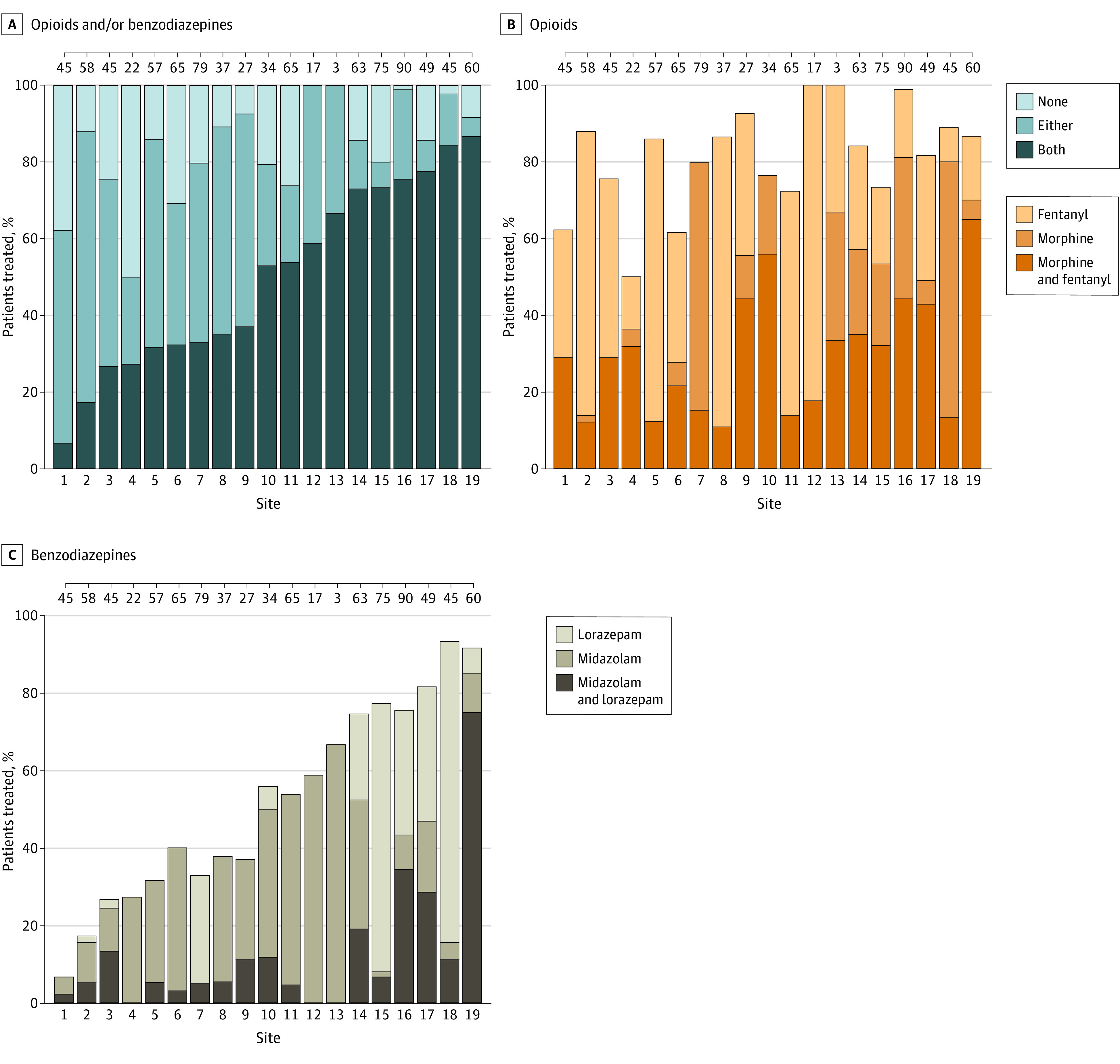
Use of Opioids and/or Benzodiazepines by Site The order of the 19 Preterm Erythropoietin Neuroprotection trial sites remains the same for the 3 panels. Ticks across upper boundary indicate the number of infants at each site.

The use of opioids and benzodiazepines varied by site, with 1 site having half of their infants (11 of 22 [50%]) without any exposure, while other sites had all infants (3 of 3 and 17 of 17) receiving at least 1 of the medications of interest during their NICU stay (Figure 2). Across the 19 sites, mean (range) opioid use was 81% (50%-100%) (mean [range], morphine: 45% [11%-81%]; fentanyl: 65% [15% to 100%]). The mean (range) use of benzodiazepines was 52% (7%-93%) (mean [range], midazolam: 37% [5%-85%]; lorazepam: 27% [0%-89%]).

### BSID-III Assessments at 2 Years’ Corrected Age

There were 692 infants (74%) with available neurodevelopmental follow-up data at 2 years’ corrected age who were included in the analysis. There were 113 infants (12%) who died, and 106 (94%) of them died prior to discharge. All 3 BSID-III subscale scores were negatively associated with the combined use of opioid and benzodiazepine exposure in this cohort, even after adjusting for potential confounding factors. The estimated difference in mean scores for infants exposed to both opioids and benzodiazepines compared with infants without exposure were −5.72 (95% CI, –8.88 to –2.57) for the cognitive scale, –8.31 (95% CI, –11.8 to –4.83) for the motor scale, and –4.47 (95% CI, –7.96 to –0.99) for the language scale.

**Figure 3.**
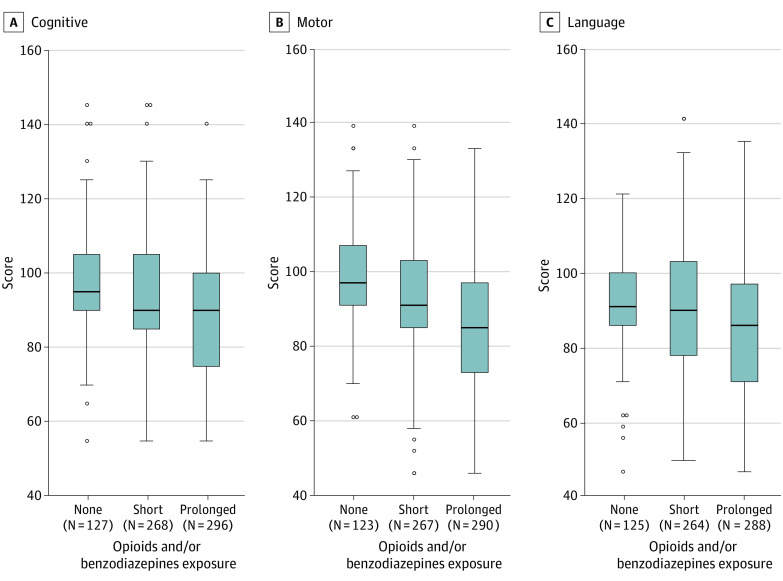
Bayley Scales of Infant Development–Third Edition (BSID-III) Cognitive, Motor, and Language Scores by Days of Exposure to Opioids and/or Benzodiazepines Box plot of median (interquartile range) cognitive, motor, and language scores at 2 years’ corrected age by exposure to opioids and/or benzodiazepines, defined as total number of days of exposure to opioids and/or benzodiazepines. Short exposure was defined as exposure to the medications of interest for 7 or fewer days; prolonged exposure, more than 7 days. Boxes contain 50% of data, with the inside horizontal line representing the median value; whiskers contain 100% of data, except for statistical outliers, which are shown as individual data points.

The median (IQR) scores varied by individual medications and length of exposure (eFigure in Supplement 1), with lower scores noted when any of the medications of interest were used for more than 7 days (Figure 3). The largest difference was noted on the BSID-III motor scores, with a median (IQR) score of 97 (91-107) in infants with no exposure compared with 85 (73-97) in infants who received opioids and/or benzodiazepines for periods longer than 7 days.

**Figure 4.**
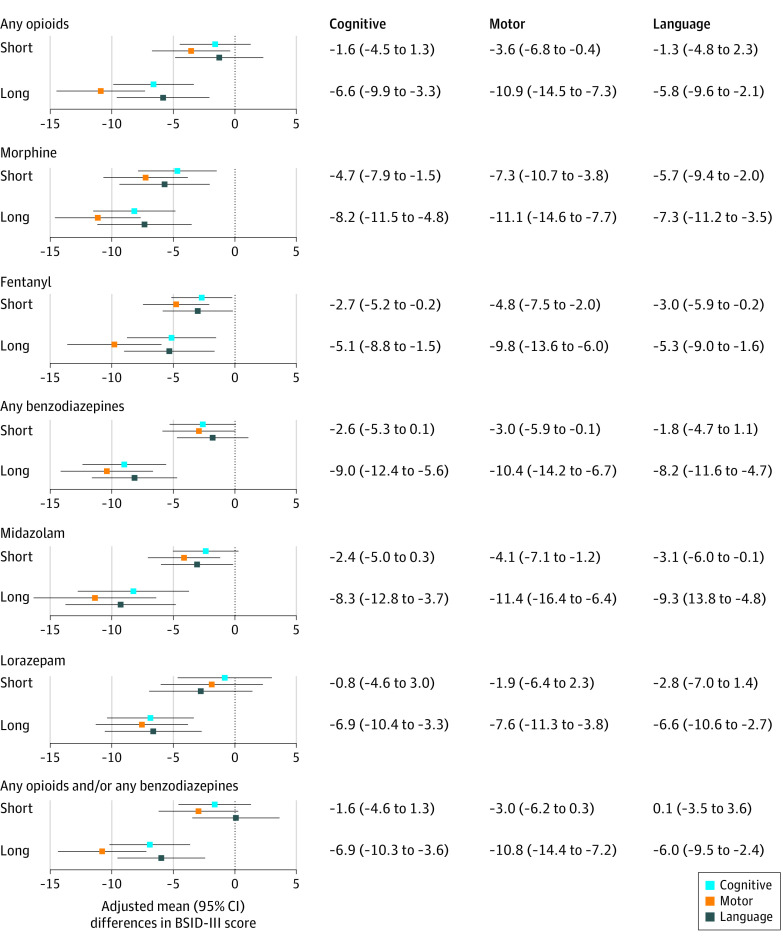
Adjusted Mean Differences in Bayley Scales of Infant Development–Third Edition (BSID-III) Cognitive, Motor, and Language Scores Comparing Infants With Specific Medication and Length of Exposure to Infants With No Exposure Adjusted differences and 95% CIs for BSID-III cognitive, motor, and language scores across infants exposed to the medications of interest by length of exposure. Short exposure was defined as exposure to the medications of interest for 7 or fewer days; prolonged exposure, more than 7 days. All estimates adjust for site and propensity scores derived for each exposure category. Propensity scores for each infant were derived by fitting a logistic regression model using exposure as the outcome and adjusting for the following potential confounders, as described in the Methods section.

BSID-III scores were also associated with the duration of exposure and class of medications (Figure 4). The estimated difference in mean scores for infants with short exposure to both opioids and benzodiazepines were –1.60 (95% CI, –4.58 to 1.31) for the cognitive scale, –2.95 (95% CI, –6.19 to 0.28) for the scale motor, and 0.09 (95% CI, –3.46 to 3.64) for the language scale. Overall, the BSID-III motor scores were lower in infants with any exposure.

## Discussion

In this study of contemporary EP infants from a multicenter clinical trial, infants with exposure to both opioids and benzodiazepines during their NICU stay were more likely to have increased in-hospital morbidities, prolonged hospitalization, and ultimately lower BSID-III cognitive, motor, and language scores compared with infants exposed to opioids or benzodiazepines alone or infants with no exposure. Prolonged exposure to any of the medications of interest (ie, morphine, fentanyl, midazolam, and lorazepam) had a negative association with all BSID-III scores at 2 years’ corrected age when compared with infants with no exposure. BSID-III scores for infants with short exposure to opioids and/or benzodiazepines were not significantly different compared with infants without exposure.

Our findings highlight the variability of exposure to opioids and benzodiazepines across NICUs and the differences between uses of the 2 classes of medications examined in this study. This is consistent with previous reports showing considerable analgesic and sedative practice variation among hospitals, even for infants with similar characteristics, illness severity, and procedural burden.^10,19^ Opioids are used commonly in the modern NICU.^20^ It is not surprising that more than half of the infants in this cohort were exposed to fentanyl and nearly half to morphine. In recent reports, fentanyl was the 7th and morphine the 14th most commonly used drug in more than 300 NICUs throughout the United States,^21^ with nearly half of infants born at 23 to 24 weeks’ gestation exposed to fentanyl and one-third to morphine.^22^

Benzodiazepines were used less frequently than opioids, with similar distribution between midazolam and lorazepam. This is consistent with data showing the decreasing use of benzodiazepines in the NICU,^10^ given the link to severe IVH, periventricular leukomalacia, or death in preterm infants.^23^

Infants included in our study who had prolonged intubation or were receiving ventilatory support at 36 weeks’ corrected age were more likely to have received opioids and/or benzodiazepines compared with other infants. Although we did not specifically evaluate the outcomes of this subgroup, clinical trials of opioid therapy used during mechanical ventilation have shown mixed results. Despite the promising results of Neonatal Outcome and Prolonged Analgesia in Neonates (NOPAIN) trial,^23^ the NEOPAIN trial^24^ demonstrated that infants randomized to preemptive analgesia with morphine had increased duration of mechanical ventilation, delayed tolerance of enteral feedings, and subtle tone abnormalities at 36 weeks’ postmenstrual age. Long-term follow-up study results were conflicting, with some impairment suggested at 5 years (ie, lower scores on the visual analysis domain of intelligence quotient), but potential subtle benefits of randomization to morphine at age 8 to 9 years (ie, superior executive functions as assessed by parent report, although no difference by teacher report or standardized assessment by the study team).^25,26,27^ The EPIPAGE study^28^ showed that very preterm infants exposed to prolonged sedation and analgesia had severe or moderate disability at 5 years (41 of 97 [42%]), more often than those who were not exposed (324 of 1248 [26%]); however, after adjusting for GA and propensity score, the authors concluded that this association was no longer statistically significant (adjusted risk ratio, 1.0; 95% CI, 0.8-1.2).^28^

Infants exposed to morphine or fentanyl had lower BSID-III scores when compared with infants with no exposure, with motor scores being the most affected. Several animal models suggest that exposure to postnatal morphine affects both motor and learning abilities.^29,30,31,32^ Similarly, infants randomized to the morphine group in the NEOPAIN trial had increased tone at term when compared with infants in the placebo group.^33^ Studies have also shown detrimental associations with fentanyl administration, including reduced cerebellar growth at term-equivalent age and an association with neurodevelopmental adverse effects at 2 years’ corrected age.^11,34^

Long-term outcomes associated with the use of benzodiazepines for this population are lacking, but preclinical data noted neuroapoptosis as well as long-term functional deficits and atypical behavioral patterns.^35^ Prolonged use of benzodiazepines was associated with lower cognitive, motor, and language scores in our cohort. Given the short-term and long-term consequences of benzodiazepines in EP infants, their use should be judicious and limited.^5^

When looking at both medication classes of interest, short-term use did not appear to significantly affect BSID-III scores, but using these medications for more than 7 cumulative days was associated with lower cognitive, motor, and language scores. Thus, it is of great concern that infants born at lower GAs are exposed to opioids and benzodiazepines for prolonged periods of time during early periods of rapid brain change and development.

### Limitations

Our study has several limitations. First, the primary randomized trial (PENUT) was not designed to definitively evaluate the impact of opioids and benzodiazepines on 2-year outcomes. Although we adjusted for known potential confounders, there may still be residual confounding by indication, and a major limitation is the lack of variables evaluating the burden of invasive ventilation and evaluation of pain and discomfort in this data set. Infants received the medications based on the attending physician’s clinical judgment, which is reflected in the different clinical characteristics of the groups. There was also wide variability in the use of opioids and benzodiazepines among the PENUT sites. A second limitation of our study is the lack of specific dosing data available. Although daily administration is important, we cannot differentiate between 1 dose in a day vs a continuous infusion, and we did not examine whether the drugs of interest were given in combination or in series. Prior studies demonstrated that the adverse effects of these medications are affected by dose, duration, and metabolism.^12,34,36,37^ Data on quantification of neonatal pain was not available for this cohort, so the potential sedative and analgesic effect of opioids and benzodiazepines could not be examined. Third, our findings do not necessarily reflect causal associations and may be due to unmeasured factors associated with exposure to analgesics and/or sedatives and infant neurodevelopmental outcomes.

## Conclusions

In this large cohort of EP infants, we found that prolonged use of opioids and benzodiazepines was associated with lower BSID-III cognitive, motor and language scores at 2 years’ corrected age. The known direct and indirect adverse effects of opioid and benzodiazepine exposure on neuronal injury and neurodevelopment must be weighed against the clear adverse effects of untreated pain and agitation on the developing brain. Standardized nonpharmacologic interventions, such as proper containment, optimizing sensory experiences, and encouraging maximal parental presence with limited pharmacologic interventions for painful procedures and intraoperative and postoperative periods may be the safest options for this population. As greater numbers of EP infants survive, it is critical to study the risks and benefits of prolonged opioid and benzodiazepine exposure balanced with painful procedures in this population with long-term follow-up studies indicated.

## Notes

Supplement 1. **eFigure.** BSID-III Cognitive, Motor, and Language Scores by Days of Exposure to Individual Medications of Interest

Supplement 2. Nonauthor Collaborators
