# Supplementary material for: Assessment of 2-Year Neurodevelopmental Outcomes in Extremely Preterm Infants Receiving Opioids and Benzodiazepines
Source: JAMA Netw Open. 2021 Jul 7;4(7):e2115998. doi: 10.1001/jamanetworkopen.2021.15998 (PMC8264640; doi:10.1001/jamanetworkopen.2021.15998)
Supplement: Supplement 1. — eFigure. BSID-III Cognitive, Motor, and Language Scores by Days of Exposure to Individual Medications of Interest [file jamanetwopen-e2115998-s001.pdf]

Supplemental Online Content

Puia-Dumitrescu M, Comstock BA, Li S, et al; PENUT Consortium. Assessment of 2-year neurodevelopmental outcomes in extremely preterm infants receiving opioids and benzodiazepines. *JAMA Netw Open.* 2021;4(7):e2115998. doi:10.1001/jamanetworkopen.2021.15998

eFigure. BSID-III Cognitive, Motor, and Language Scores by Days of Exposure to Individual Medications of Interest

This supplemental material has been provided by the authors to give readers additional information about their work.

© 2021 Puia-Dumitrescu M et al. *JAMA Network Open.*

eFigure. BSID-III Cognitive, Motor, and Language Scores by Days of Exposure to Individual Medications of Interest

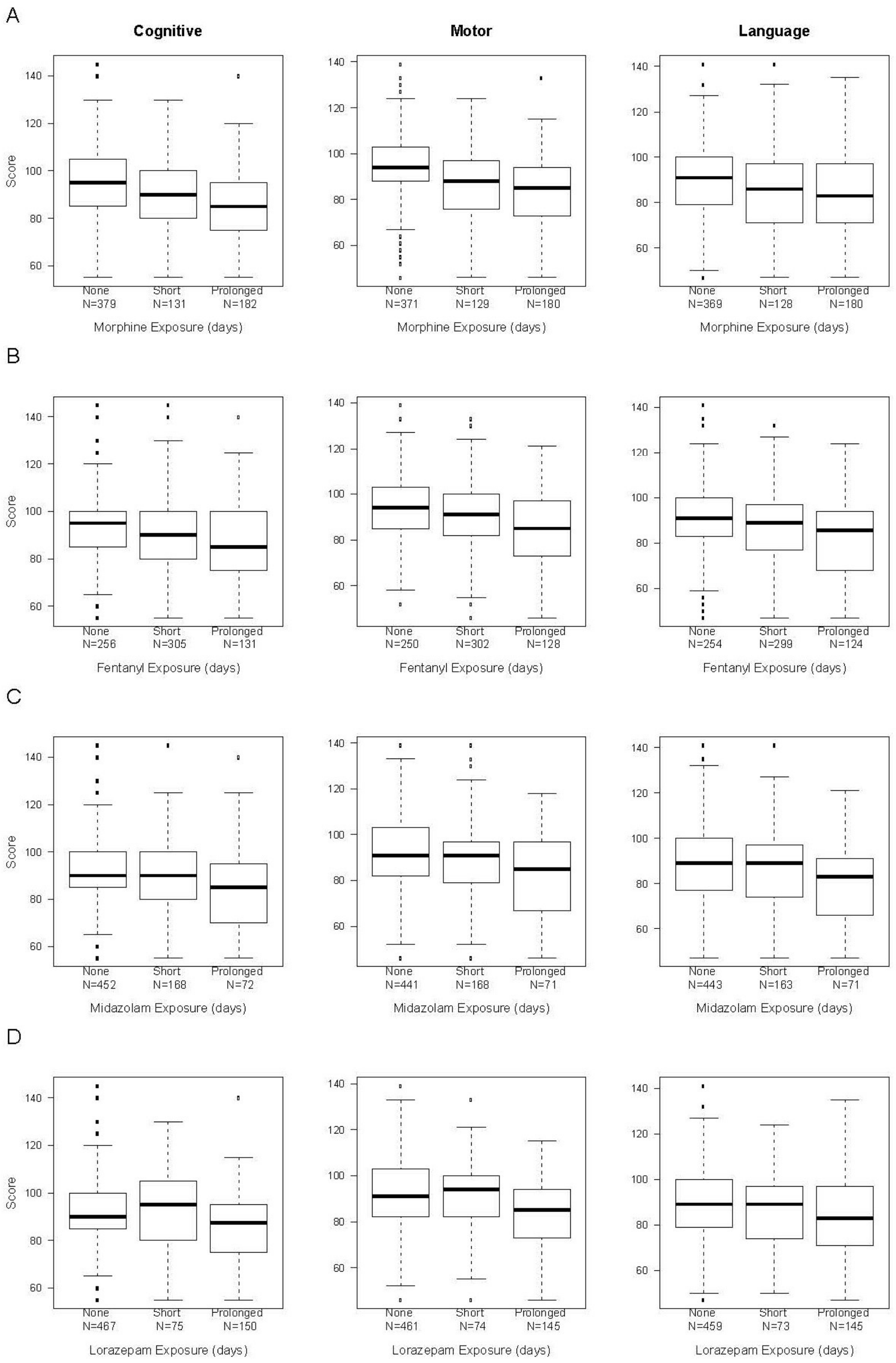

© 2021 Puia-Dumitrescu M et al. *JAMA Network Open.*

Boxplot of median (IQR) cognitive, motor and language scores at 2 years' corrected age by exposure to opioids and/or benzodiazepines defined as total number of days of exposure to morphine (A), fentanyl (B), midazolam (C) and lorazepam (D). Short exposure was defined as exposure to the medications of interest for 7 or fewer days, and prolonged exposure for exposure more than 7 days. Boxes contain 50% of data with the inside horizontal line representing the median value; whiskers contain 100% of data, except for statistical outliers shown as individual data points.

© 2021 Puia-Dumitrescu M et al. *JAMA Network Open.*
